# Multimodal deep learning-based diagnostic model for BPPV

**DOI:** 10.1186/s12911-024-02438-x

**Published:** 2024-03-21

**Authors:** Hang Lu, Yuxing Mao, Jinsen Li, Lin Zhu

**Affiliations:** State Key Laboratory of Power Transmission Equipment Technology, School of Electrical Engineering, Chongqing University, Chongqing, China

**Keywords:** Vertigo, BPPV, Deep learning, Multimodal, Feature fusion

## Abstract

**Background:**

Benign paroxysmal positional vertigo (BPPV) is a prevalent form of vertigo that necessitates a skilled physician to diagnose by observing the nystagmus and vertigo resulting from specific changes in the patient’s position. In this study, we aim to explore the integration of eye movement video and position information for BPPV diagnosis and apply artificial intelligence (AI) methods to improve the accuracy of BPPV diagnosis.

**Methods:**

We collected eye movement video and diagnostic data from 518 patients with BPPV who visited the hospital for examination from January to March 2021 and developed a BPPV dataset. Based on the characteristics of the dataset, we propose a multimodal deep learning diagnostic model, which combines a video understanding model, self-encoder, and cross-attention mechanism structure.

**Result:**

Our validation test on the test set showed that the average accuracy of the model reached 81.7%, demonstrating the effectiveness of the proposed multimodal deep learning method for BPPV diagnosis. Furthermore, our study highlights the significance of combining head position information and eye movement information in BPPV diagnosis. We also found that postural and eye movement information plays a critical role in the diagnosis of BPPV, as demonstrated by exploring the necessity of postural information for the diagnostic model and the contribution of cross-attention mechanisms to the fusion of postural and oculomotor information. Our results underscore the potential of AI-based methods for improving the accuracy of BPPV diagnosis and the importance of considering both postural and oculomotor information in BPPV diagnosis.

## Background

In recent years, deep learning methods have been widely used in the field of medical image processing [1–5]. For the diagnosis of many types of diseases, multiple forms of data are often required to be considered together, such as textual information (clinical presentation, past medical history, blood and urine test indicators), images (MRI, CT, ECG, etc.), etc. Traditional deep learning methods can usually only input a single form of data (time series, pictures, videos), which will be difficult to apply to the medical diagnosis that integrates many forms of data for consideration. In contrast, in recent years, research on multimodal fusion techniques in deep learning has progressed rapidly [6, 7] and its application in medical diagnosis has attracted widespread attention [8, 9]. The difficulty of how to apply deep learning methods to the diagnosis of BPPV lies in how to combine eye movement information (video)with head position information (vector). Accordingly, we propose a BPPV diagnosis model based on a multimodal deep learning approach. In this study, we collaborated with relevant hospitals to collect eye movement videos and postural information from 518 BPPV patients. We propose a multimodal deep learning-based BPPV diagnostic model, consisting of three parts: eye movement feature extraction, postural vector encoding, and feature fusion using a cross-attention mechanism module. Our proposed model achieves good performance on our acquired BPPV dataset, demonstrating its effectiveness for diagnosing BPPV.

BPPV is one of the most common vestibular peripheral disorders. Its prevalence is high, accounting for approximately one quarter of clinical vertigo patients, with a lifetime prevalence of 2.4%, a cumulative population prevalence of up to 10%, and a recurrence rate of 50. Concerning this type of disorder, the annual incidence rate in the population aged 60 and above is approximately 3.4%, with a higher incidence rate in females compared to males [10–12]. To diagnose BPPV, nystagmus analysis in different positions is commonly used [11, 13, 14], and there have been several relevant studies in this regard. For example, a lateral roll test used for BPPV diagnosis can conveniently determine the affected ear [15]; some studies have found that patients, in specific test positions, undergo nystagmus in a particular direction [16]; and there is a BPPV-assisted diagnosis and treatment system that transforms manual consultation into automatic consultation, incorporating crucial neurological otologic examinations such as the Dix-Hallpike maneuver and Roll test [17]. Given that BPPV diagnosis relies on eye-movement videos, deep learning methods have mainly been applied to images, texts, and temporal sequences, with less research conducted on video data and even less on the intelligent diagnosis of BPPV. Therefore, our work represents the attempt to apply deep learning methods to the diagnosis of BPPV. In the field of video understanding, the use of deep learning methods has greatly improved model accuracy in recent years. Videos are rich sources of spatio-temporal information, and models need to effectively represent this information. The multi-input channel network structure in 2DCNN(Convolutional Neural Network), which takes spatial and temporal information as separate inputs, has been commonly used for this purpose [18].

Additionally, some models directly take video data as input and automatically extract temporal information using frame differences within the model [19]. With 3DCNN, three-dimensional convolutional kernels can effectively integrate spatio-temporal information, replacing the two-dimensional convolutional kernels of the 2DCNN structure [20]. By designing a two-channel structure, where each 3DCNN channel focuses on learning spatial and temporal information, respectively, the model can further improve performance [21]. To automatically retrieve the optimal 3DCNN structure, a neural network search algorithm can be used in combination with other algorithms [22, 23]. Vit(Vision in Transformer) has shown significant potential in the image field [24], and the self-attention mechanism, which effectively integrates spatio-temporal information, can be a valuable tool for video analysis [25]. Recent studies have explored the potential of CNNs in feature extraction [26, 27], with larger convolution kernels that have larger perceptual fields than the typical 3x3 convolution kernels being especially helpful for improving model performance. In the context of diagnosing BPPV, eye-movement feature information contained in eye-movement videos is crucial. To enable intelligent diagnosis of BPPV, CNNs with large convolution kernels that can effectively extract spatio-temporal information are needed.

Multimodal learning is the capability of a model to reason, learn, and understand information across different modalities. It achieves this by enabling the interaction and transformation of each modality, leading to the synthesis and analysis of multiple modalities for decision-making. The remarkable success of deep learning models such as CNNs and Transformers with diverse types of data, including images, text, and sequences, attests to their aptitude in comprehending multiple modalities and their deployment in multimodal learning [28, 29]. Feature fusion from different modalities is achieved through an encoder-decoder structure, which enables interaction for multimodal information integration, subsequently inputting to a classifier for classification [30]. Cross-attention, on the other hand, demonstrates a superior ability to interactively fuse multimodal feature information in visual language models [31], with its promising performance in medical diagnoses such as Alzheimer’s disease and other illnesses [8].

Diagnosis of BPPV entails the combination of head position and eye movement characteristics observed from the eye movement video, which implies that BPPV diagnosis is also multimodal. By simulating the reasoning process of actual doctors during diagnosis, and considering the features of both eye movement and head position, we can achieve intelligent diagnosis of BPPV through multimodal learning.

## Methods

### Data source

**Fig. 1:**
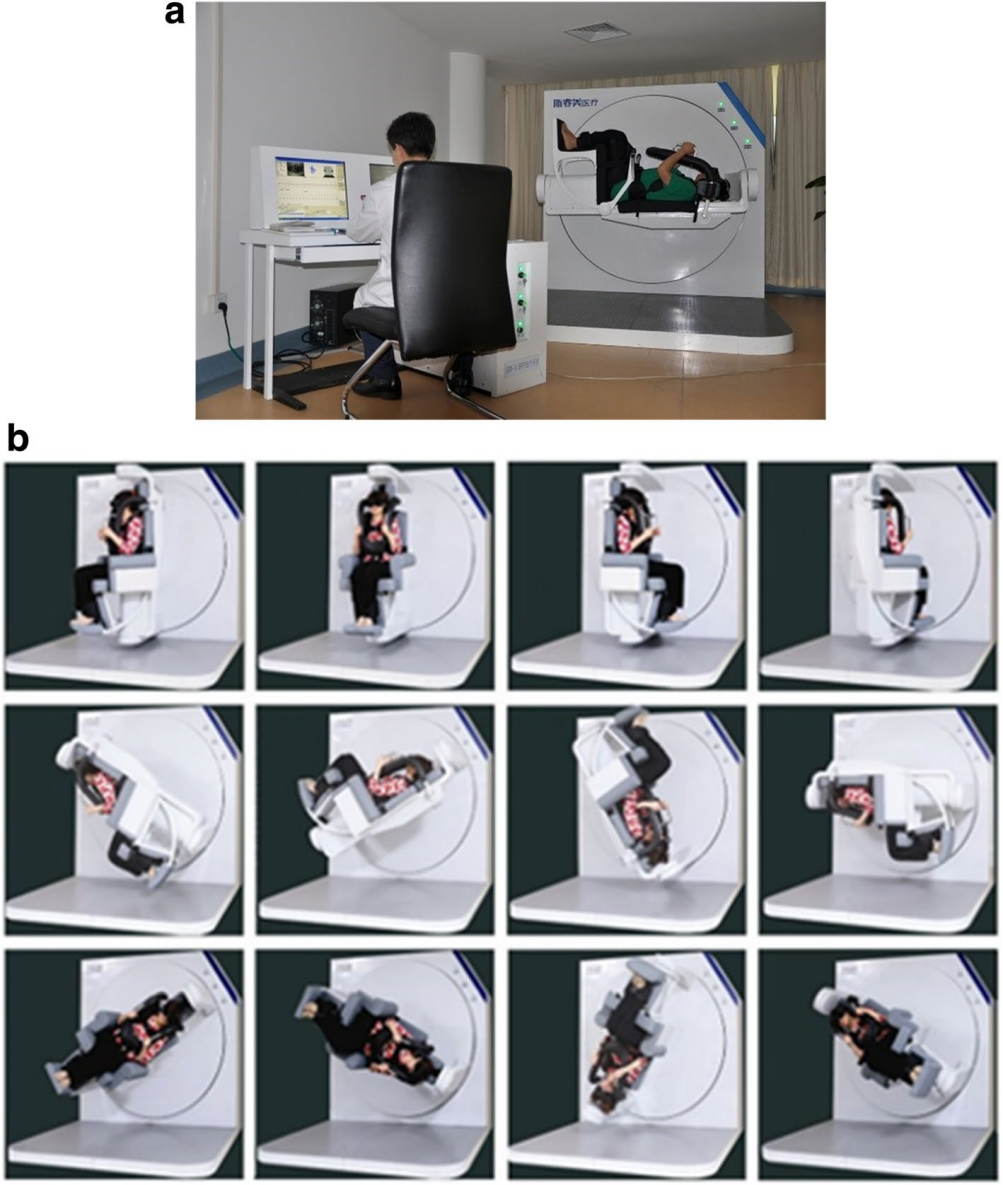
BPPV therapeutic instrument. **a**: operation scene. **b**: the treatment instrument can make the head position reach any angle and orientation through the cooperation of spindle and auxiliary axis

In collaboration with the Second Affiliated Hospital of Army Medical University(Xinqiao Hospital), we collected eye movement videos from the hospital’s vertigo clinic from January 2021 to March 2021 from 518 BPPV patients who came to the hospital for examination during this period and their diagnostic findings. Also, during the collection process, only the examination number is retained and no data related to the patient’s personal information is collected. The study methods and data collection were reviewed by Xinqiao Hospital’s ethics committee, which waived the application for informed consent. The data is collected from hospitals utilizing BPPV diagnostic instruments. As shown in Fig. 1, the diagnostic instruments are equipped with two rotation axes - the main axis and the auxiliary axis - which allow the patient to be positioned at any angle. By following a specific test protocol, a physician can adjust the patient’s position and observe their eye movements to draw a diagnostic conclusion [12].

These cases were divided into six categories based primarily on the location of the otolith in the semicircular canal: left posterior canal, right posterior canal, left horizontal canal, right horizontal canal, cupulolithiasis and cured or asymptomatic,The number of cases within each of these categories is shown in Table 1. We randomly split the dataset into training and test sets in an 8:2 ratio in each category.

**Table 1:** Number of BPPV cases of each type in the dataset

BPPV Types	Quantity
Left posterior canal	120
Right posterior canal	147
Left horizontal canal	52
Right horizontal canal	81
Cupulolithiasis	67
Cured or asymptomatic	51

### Preprocessing

**Fig. 2:**
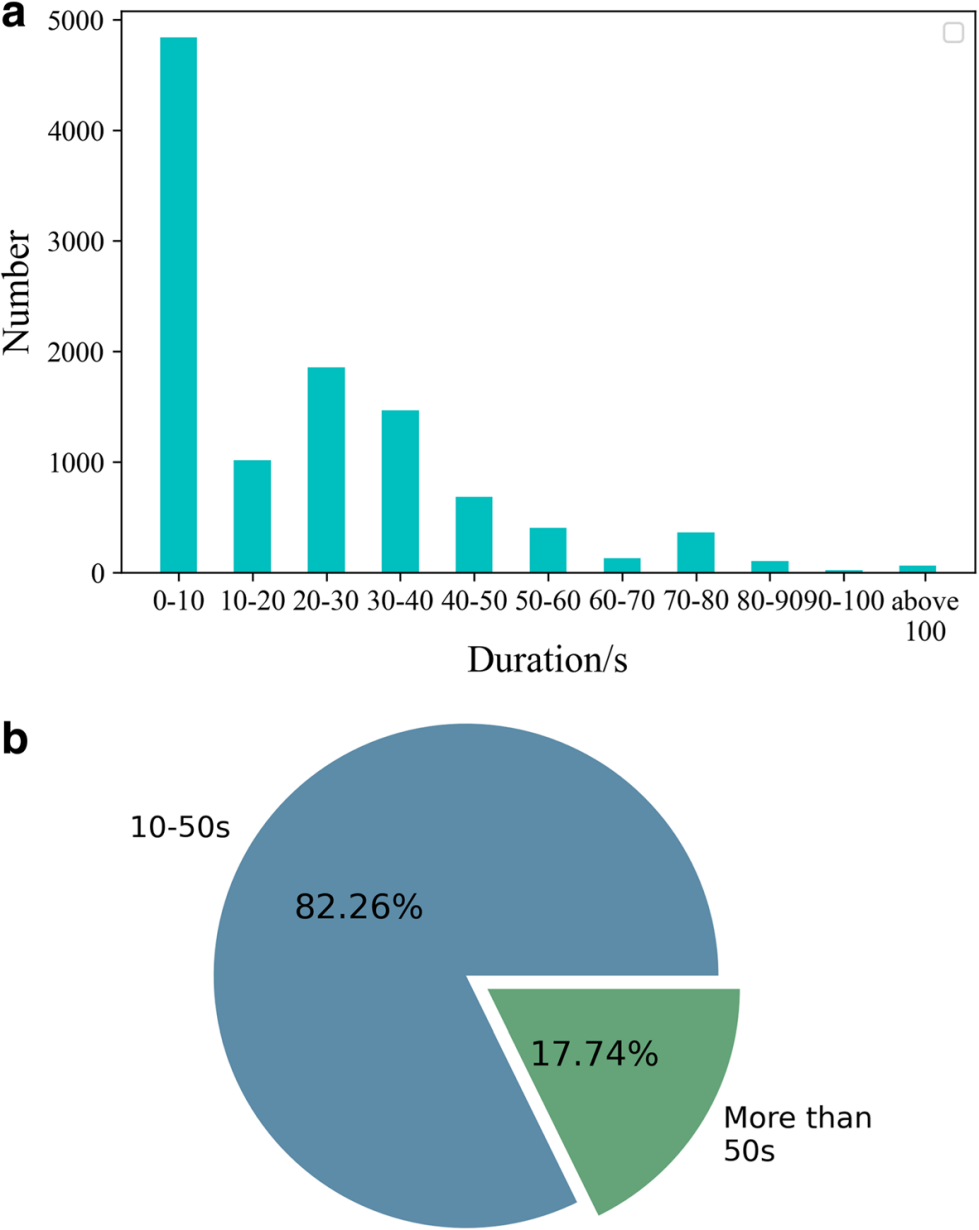
**a**: the eye-movement video statistics of each duration. **b**: the percentage of videos of duration 10-50s and 50s or more, excluding videos of duration 0-10s

**Fig. 3:**
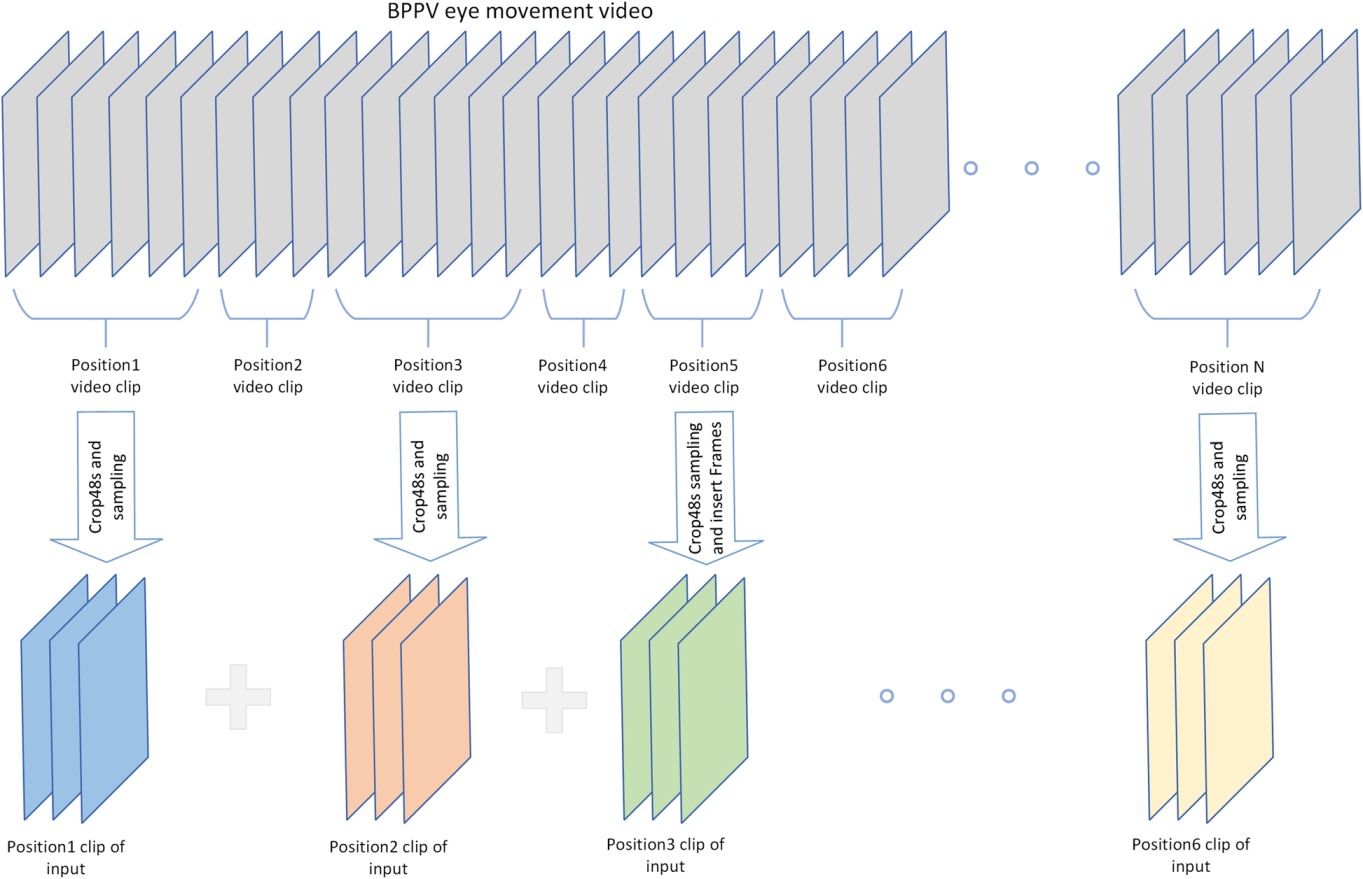
Video sampling process. Video clips less than 10s in length, such as the head position 2 clips in the figure, we do not use them as input, some clips do not reach 48s in length, we interpolate them to make their length consistent

The length of eye movement videos varies across patients in our dataset. Patients with cupulolithiasis may experience prolonged episodes of nystagmus and vertigo, while those with horizontal canal BPPV may experience shorter episodes. Consequently, physicians typically keep patients in a fixed position for a set period of time during diagnosis to observe the presence of vertigo and nystagmus. However, this approach results in inconsistencies in the total length of observation and the duration of observation for specific positions, which compromises the uniformity of input necessary for model training. Additionally, the sequence of postural changes performed by physicians during diagnostic tests is standardized, with eye movements recorded for each posture in video format. However, in practice, physicians typically only observe a subset of the postures, with most positions being skipped. Statistical analysis of our dataset revealed that the majority of patients were observed in a particular posture for less than 50 seconds, and that the most observation time was between 10 and 50 seconds, with fewer than six postures requiring longer observation times. The shorter observation times, typically less than 10 seconds, include time for position changes and for the physician to attend to the patient’s comfort, and are thus unlikely to contain useful information. Figure 2 shows the resulting statistics. To address this issue, we employed a preprocessing strategy to standardize the input: we selected six video segments from postures with longer observation times totaling 48 seconds, resulting in 1200 frames of video. After sampling the video at equal intervals and selecting one frame out of every four, we obtained a 300-frame video. For postures where the video was shorter than 48 seconds, we inserted black images with zero pixel values to align the image matrix. We then combined six video segments to produce a total of 1800 frames of input data. Figure 3 illustrates this approach.

### Model structure

**Fig. 4:**
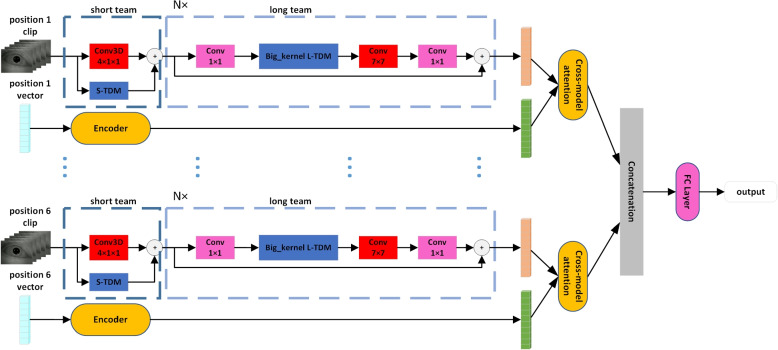
Model structure. In the short-term module, the convolutional kernel is a 4$\times$1$\times$1 3D convolutional module for downsampling in the time dimension. In the big kernel long-term module, the large convolution kernel expands the model perceptual field and extracts eye-movement features

The association between a video and its corresponding head position vectors in each head position can be difficult for a model to learn when the video contains six head positions and six head position vectors simultaneously. To address this challenge, we process the videos segment by segment and utilize a video understanding module in our model to output six dynamic feature vectors, each of which corresponds to an eye-movement video recorded in a specific head position. We then feed the corresponding head position vectors of the six head positions to an Encoder to output the six head position feature vectors. In Fig. 4, we demonstrate that the resulting eye-movement feature vectors and head position feature vectors are input into a cross-attention module for information cross-fusion, ultimately generating fused feature vectors. These vectors are stitched together across various head positions, and a classifier is selected from a layer of FC(Fully Connected) layer to classify the fused feature vectors.

#### Head position vector self-encoder

In medical diagnosis, accounting for head position is a crucial aspect as it describes the patient’s current position in terms of upright, supine, and lateral positions. To intuitively represent postural information, we normalize the rotation angles of the major and minor axes (angles in the range of (-360,360)) to form a two-dimensional vector as the postural vector. To make the head position information contained in the head position vector learnable by the model, we propose adding a head position vector self-encoder. The self-encoder abstracts the two rotation axis angles into head position feature vectors. The encoder part of the self-encoder comprises three-layer FC layers, while the decoder part comprises two-layer FC layers.

Moreover, we designed a pre-training task to simulate the direction of the human eye’s line of sight in 3D coordinates for each head position during the actual diagnosis. We utilize the main axis and auxiliary axis angles as variables, and the decoder outputs its coordinates in the 3D Cartesian coordinate system (see equation 12). Finally, the mean square error between the output of the decoder and the actual transformed 3D Cartesian coordinates is added to the final loss function as a penalty term. This way, the self-encoder can learn the spatial information contained in the head position vector during the model training process.

#### Eye movement feature extraction

**Fig. 5:**
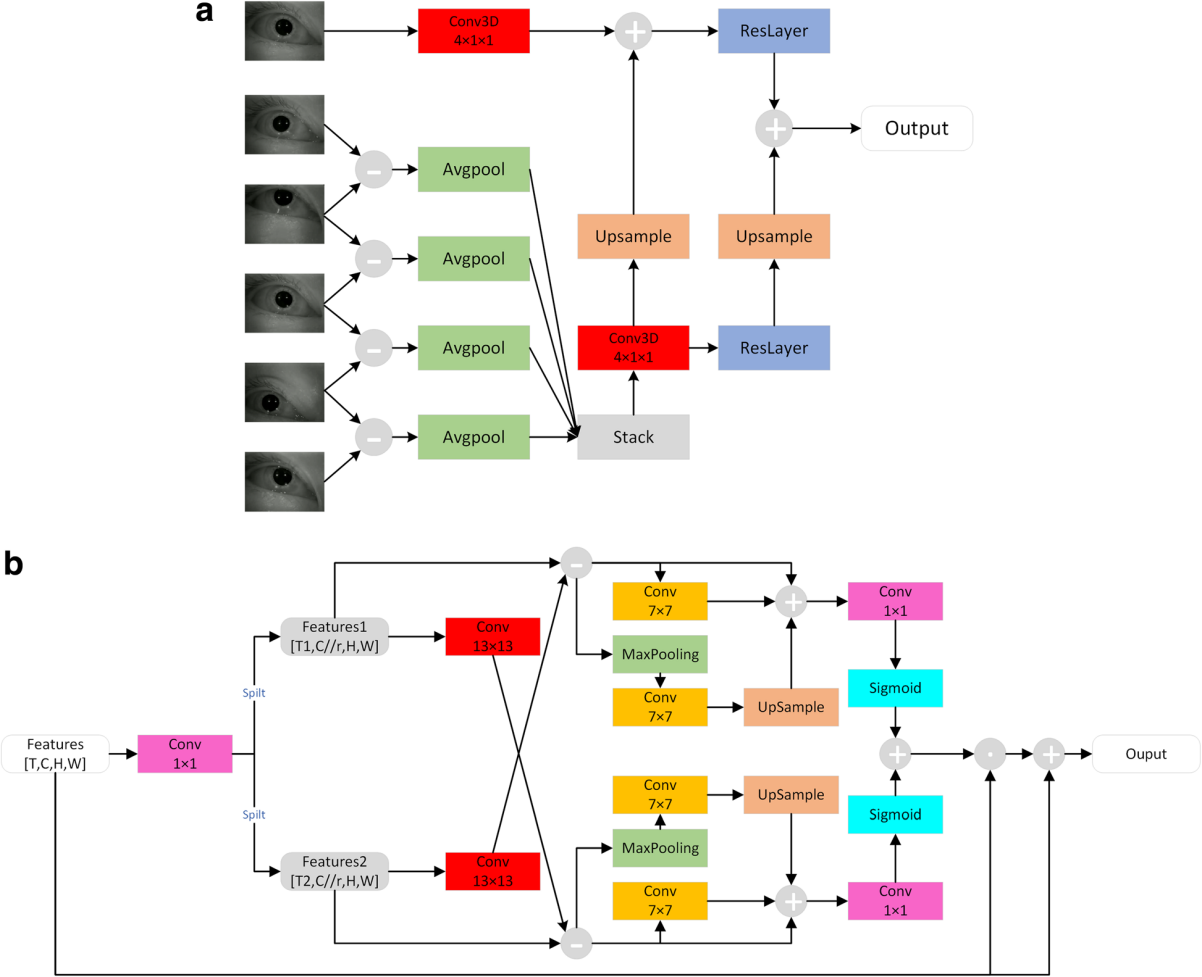
BKTDN structure. **a**: short-term module structure **b**: Big kernel long-term module structure

The eye-movement video dataset of BPPV, composed of simple scenes, objects, and movements, presents a unique challenge. The patient wears a helmet during the detection and thus the background part of the video is black, with the only object being the patient’s eye. Movement is restricted to the eye part, with the representation of the area other than the eye on the image being essentially unchanged. As a result, the amount of information contained in this type of video is relatively sparse. However, due to the need to observe in multiple positions, and for some types of BPPV (e.g., Cupulolithiasis), a certain amount of time in a specific position is required. Thus, post-preprocessing, the video length is long, with 1800 frames per case. We posit that the eye-movement video dataset has two distinct features: 1) the scenes are single and the information content is relatively sparse; 2) the video length is long, offering a wealth of data. To address these features, we employ TDN(Temporal Difference Module), a lightweight video understanding model based on 2D convolutional neural networks, as the eye-movement feature extraction model [19]. TDN comprises two modules: a short-term and a long-term module, and details on both modules can be found in Fig. 5a and b

The Short-term module utilizes a frame difference approach and extracts motion features via the residual network layer (ResLayer) [32] in a low-resolution architecture to minimize the number of model parameters. Despite being restricted to a lower resolution, this architecture does not significantly sacrifice accuracy and is particularly well-suited for the BPPV eye-tracking dataset. Although the original architecture introduces a downsampling process, it does not take the temporal dimension into account. As the video length of the BPPV eye-movement dataset is larger than most public datasets, valid information is mainly concentrated in the period before and after the occurrence of nystagmus; thus, the temporal dimension exhibits a sparse signal. In light of this, we replaced the 2D convolution operation in the original architecture of short-term with 3D convolution to downsample from the temporal dimension, drastically reducing the computational effort. The output flow of short-term module is as follows:

1 $$\begin{aligned} \text {O}_{1}=Conv3D(Avgpool(D(I_i)))\end{aligned}$$

2 $$\begin{aligned} \text {O}_{2}=Conv3D(I_i)+Upsample(O_1)\end{aligned}$$

3 $$\begin{aligned} \text {Output}=Reslayer(O_1)+Upsample(Reslayer(O_2)) \end{aligned}$$

Where $\text {I}_{\text {i}}$ is the $\text {i}$-th frame, $\text {D}(\text {I}_{\text {i}})=[D_{-2},D_{-1},D_1,D_2]$ is the dynamic feature map obtained by frame difference between $\text {I}_{\text {i}}$ and the two frames before and after respectively. The dynamic feature map is downsampled by Avgpool averaging and 3D convolution downsampled image format of the $\text {i}$-th frame and summed to output a spatio-temporal feature map with spatial and dynamic information.

From the spatial dimension, the eye region in the eye movement image occupies a substantial portion of the image, and when the eye moves, a significant part of the image is altered. Utilizing large convolution kernels can enhance the receptive field of the convolutional layer, thus increasing the model’s capacity to detect the eye part motion. In the long-term structure, we expand the original 3$\times$3 convolutional layers into 13$\times$13 and 7$\times$7 convolutions respectively, thereby improving the model’s ability to perceive the eye movement region of the eye movement video. We name the improved long-term module as “Big kernel long-term module”. And for the whole TDN model, we named BKTDN.

4 $$\begin{aligned} \text {F}^{\text {T,C,H,W}}\xrightarrow {Conv1\times \text {1},spilt}F_{1}^{{{\text {T}}_{1}},C//r,H,W},F_{2}^{{{\text {T}}_{2}},C//r,H,W} \end{aligned}$$

5 $$\begin{aligned} \text {D}F({{F}_{1}},{{F}_{2}})={{F}_{1}}-Con{{v}_{13\times 13}}({{F}_{2}}) \end{aligned}$$

6 $$\begin{aligned} \text {M}_{1}({{F}_{1}},{{F}_{2}})=Con{{v}_{7\times 7}}(DF({{F}_{1}},{{F}_{2}}) \end{aligned}$$

7 $$\begin{aligned} \text {M}_{2}({{F}_{1}},{{F}_{2}})=Upsample(Con{{v}_{7\times 7}}(Maxpool(DF({{F}_{1}},{{F}_{2}})))) \end{aligned}$$

8 $$\begin{aligned} \text {M}({{F}_{1}},{{F}_{2}})=Sigmoid(Conv(D({{F}_{1}},{{F}_{2}})+{{M}_{1}}({{F}_{1}},{{F}_{2}})+{{M}_{2}}({{F}_{1}},{{F}_{2}}))) \end{aligned}$$

9 $$\begin{aligned} \text {O}utput=F\odot \frac{1}{2}(M({{F}_{1}},{{F}_{2}})+M({{F}_{2}},{{F}_{1}}))+F \end{aligned}$$

To obtain the long-term dynamic features, TDN splits the whole feature map into several segments of longer length, and each adjacent segment is made to differ to obtain the dynamic feature map between segments. As in Eq. 4. $\text {F}$ is the spatio-temporal feature map output by the short-term module with the length of $\text {T}$ frames $\text {F}_{1},\text {F}_{2}$ is the feature map split by channel downsampling $T\le {{T}_{1}}+{{T}_{2}}$ and ${{T}_{1}}={{T}_{2}}$.

#### Feature fusion module

The improved cross attention module based on self attention has shown good results in information fusion used for multimodal [33, 34]. We used the cross attention module for the fusion of head position information and eye movement information. Where Attention is calculated as follows.

10 $$\begin{aligned} \text {A}ttention(Q,K,V)=softmax(\frac{Q{{K}^{T}}}{\sqrt{{{d}_{k}}}})\odot V \end{aligned}$$

**Fig. 6:**
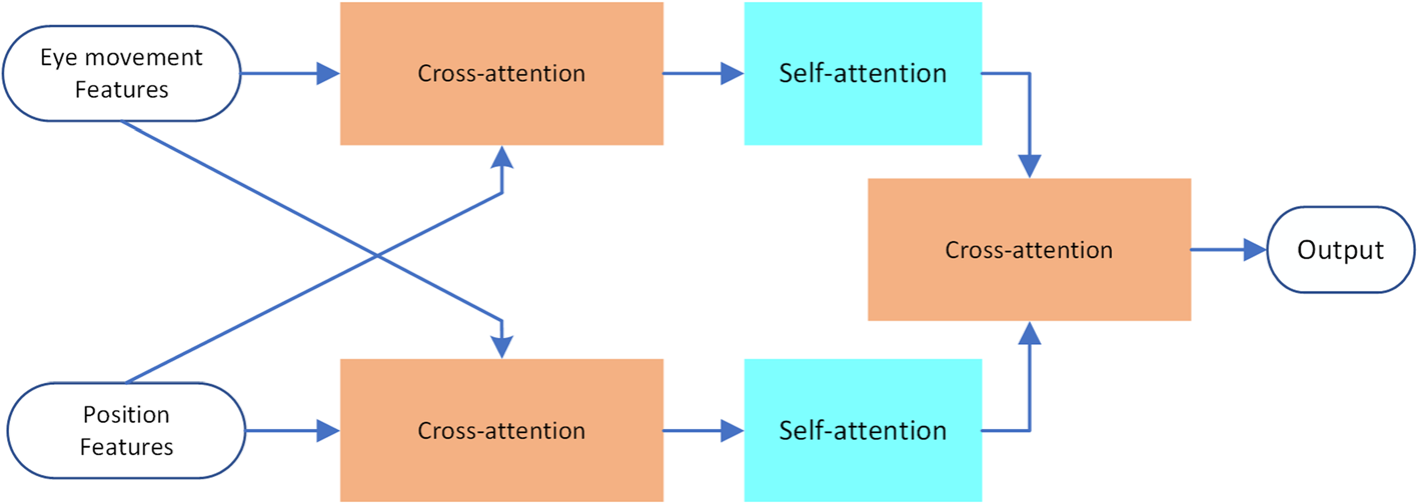
Big kernel long-term module structure. Video clips less than 10s in length, such as the head position 2 clips in the figure, we do not use them as input, some clips do not reach 48s in length, we interpolate them to make their length consistent

Specifically, after encoding the head position vector and extracting features using TDN, we obtained length-aligned head position and eye movement feature vectors. Inspired by LXMERT [31], we treated these two vectors as Query in the Cross-attention module to enable information interaction fusion. To further enhance the internal association of the fused information, we added a layer of self-attention, the structure can be seen in Fig. 6. In the end, we included another layer of cross-attention to achieve additional information interaction and improve information fusion. The calculation process is as follows:

11 $$\begin{aligned} \text {O}utput={{A}_{cross}}({{A}_{self}}({{A}_{cross}}({{F}_{e}},{{F}_{p}})),{{A}_{self}}({{A}_{cross}}({{F}_{p}},{{F}_{e}})) \end{aligned}$$

where $F_e,F_p$ refers to eye movement feature vector and head position feature vector, ${{A}_{cross}}({{F}_{e}},{{F}_{p}})$ refers to attention computation with $F_q$ as Query and $F_e$ as key and value key-value pairs. $A_{self}$ means self-attention computation.

Cross-attention is a powerful technique for fusing head position and eye movement information. In our subsequent experimental evaluation, we compared the performance of different information fusion schemes, and the experimental results demonstrated the superior performance of our proposed Cross-attention approach.

## Results

### Training strategy

In order to ensure that the encoder part of the self-encoder can learn the spatial information contained in the head position vector and encode it into a feature vector containing spatial information, we pre-trained the encoder and decoder + fine-tuned them. The pre-training task we designed is the task of simulating the orientation of the human eye’s vision, converting the principal and secondary axis angles, into coordinates in 3D space.

12 $$\begin{aligned} \left\{ \begin{array}{l} x=\cos \phi \\ y=\sin \theta \pm \sqrt{\frac{1-{{\cos }^{2}}\phi }{1+{{\tan }^{2}}\theta }} \\ z=\cos \theta \pm \tan \theta \sqrt{\frac{1-{{\cos }^{2}}\phi }{1+{{\tan }^{2}}\theta }} \\ \end{array}\right. \theta ,\phi \in (-2\pi ,2\pi ) \end{aligned}$$

**Fig. 7:**
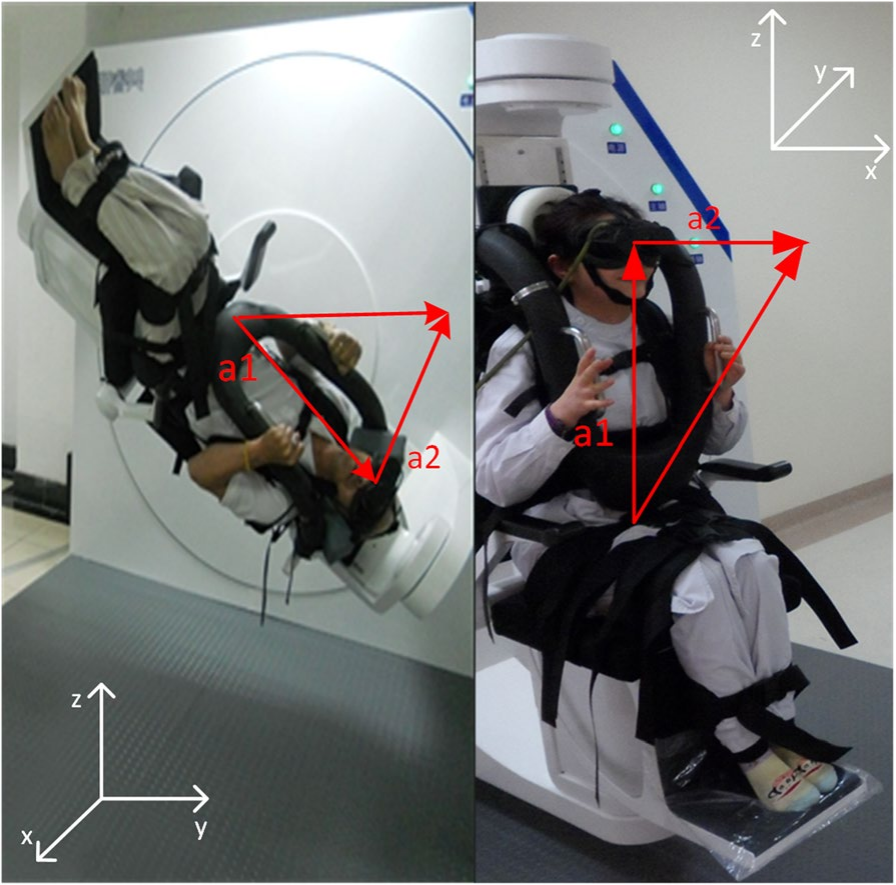
Line of sight orientation vector. The virtual vector a1 simulates the main axis, a2 simulates the auxiliary axis orientation, and the synthetic vector of a1, a2 simulates orientation of human eye

**Fig. 8:**
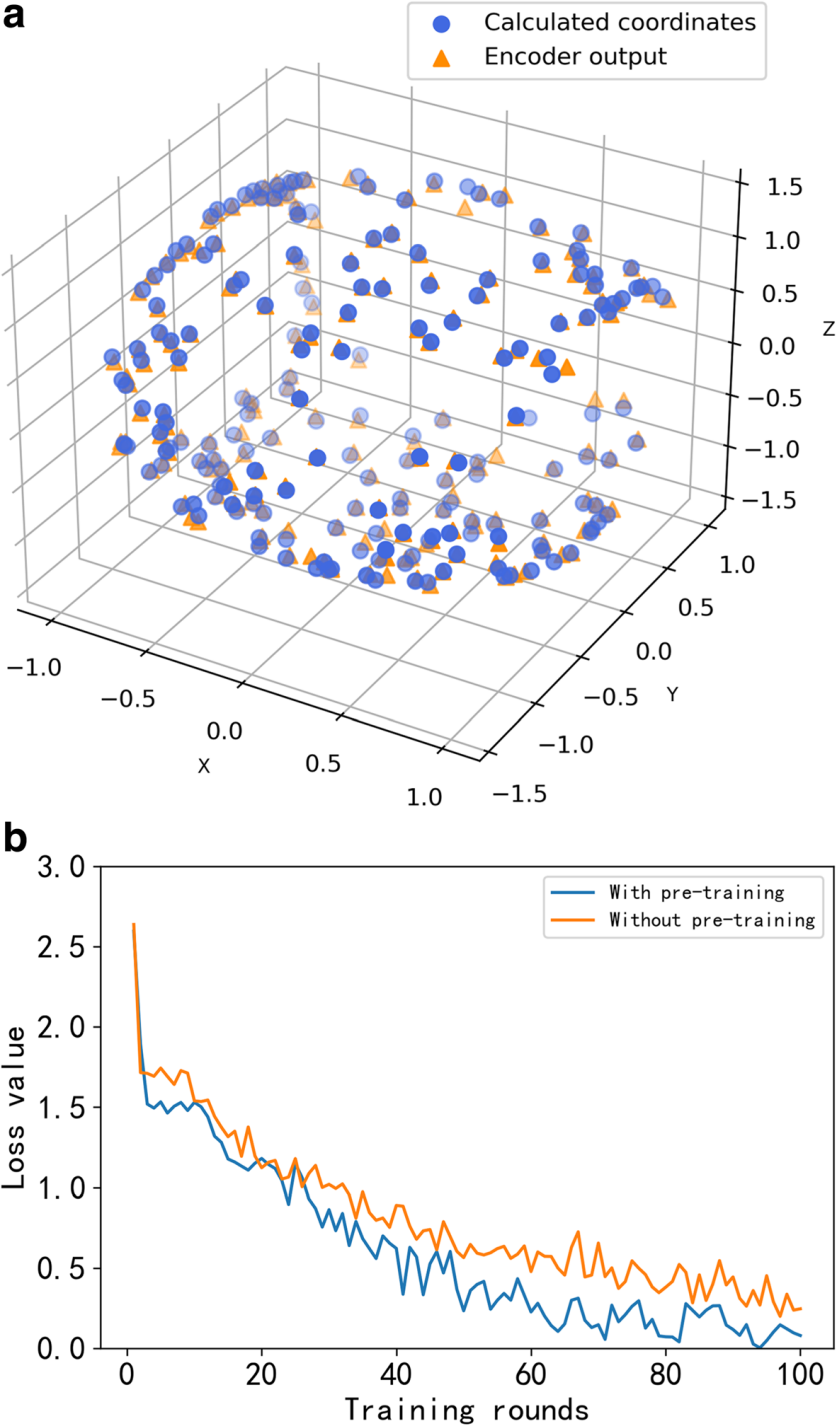
**a**: Fit of the encoder output value to the calculated value of the coordinates **b**: Comparison of the change in loss value between the training process of the model with and without encoder pre-training

We use the synthetic vector of the two vectors, which we refer to as the spatial coordinates of the line-of-sight orientation vector, to simulate the patient’s line-of-sight orientation at the time of diagnosis, as shown in Fig. 7. The spatial coordinates of the visual orientation vector are determined by the main and auxiliary axis angles, as given by Equation. To learn the spatial information in the head position vector, namely the main and auxiliary axis angles, we trained a self-encoder using the coordinates of the line-of-sight orientation vector as the output labels, and randomly generated 200 head position vectors for pre-training. After 100 rounds of training, the MAE(Mean Absolute Error) of the transformation of the line-of-sight orientation coordinates reached 0.0066. Figure 8a shows the fit of the encoder output value to the calculated value of the coordinates. Next, we loaded the pre-trained model parameters into the self-encoder in the model for formal training.

During formal training, we used the mean square error of the decoder output of the self-encoder with respect to the eye angle orientation coordinates as the penalty term of the loss function, to ensure that the training direction of the encoder is not shifted. Additionally, for the overall training of the model, we trained for 1000 rounds with a learning rate of = 0.01, using a cross-entropy function as the loss function. The variation of the loss function during the training process is shown in Fig. 8b, which demonstrates that the model converges significantly faster after the addition of the pre-trained encoder to the formal training process.

### Experimental results

To assess the individual impact of each module in our model on its overall performance, we conducted a series of separate comparison experiments. Firstly, we evaluated the diagnostic accuracy of the model using unimodal inputs and multimodal inputs, respectively, for BPPV disorders. Secondly, we investigated the feature information fusion component of the model’s multimodal data. To test the effectiveness of our cross-attention mechanism for feature fusion, we devised multiple feature fusion schemes for comparison, and evaluated their impact on the model’s performance. Finally, we introduced a large convolution kernel to adapt to the BPPV dataset, and assessed its impact on enhancing the model’s performance. The experimental results are shown in Table 2.

**Table 2:** Experimental results of the model on the BPPV test set

Model	Input	Feature fusion	Accuracy
BKTDN	Multimodal	Cross-attention module	81.7%
BKTDN	Unimodal	Self-attention module	52.8%
BKTDN	Unimodal	Concatenate	39.4%
BKTDN	Multimodal	Self-attention module	79.8%
BKTDN	Multimodal	Concatenate	73.0%
TDN	Multimodal	Cross-attention	79.8%
TDN	Multimodal	Self-attention	75.9%
TDN	Multimodal	Concatenate	71.1%
BKTDN	Multimodal	Weighted summation	73.0%
BKTDN	Multimodal	Weighted summation+Self-attention	76.9%

#### Unimodal and Multimodal Inputs

**Fig. 9:**
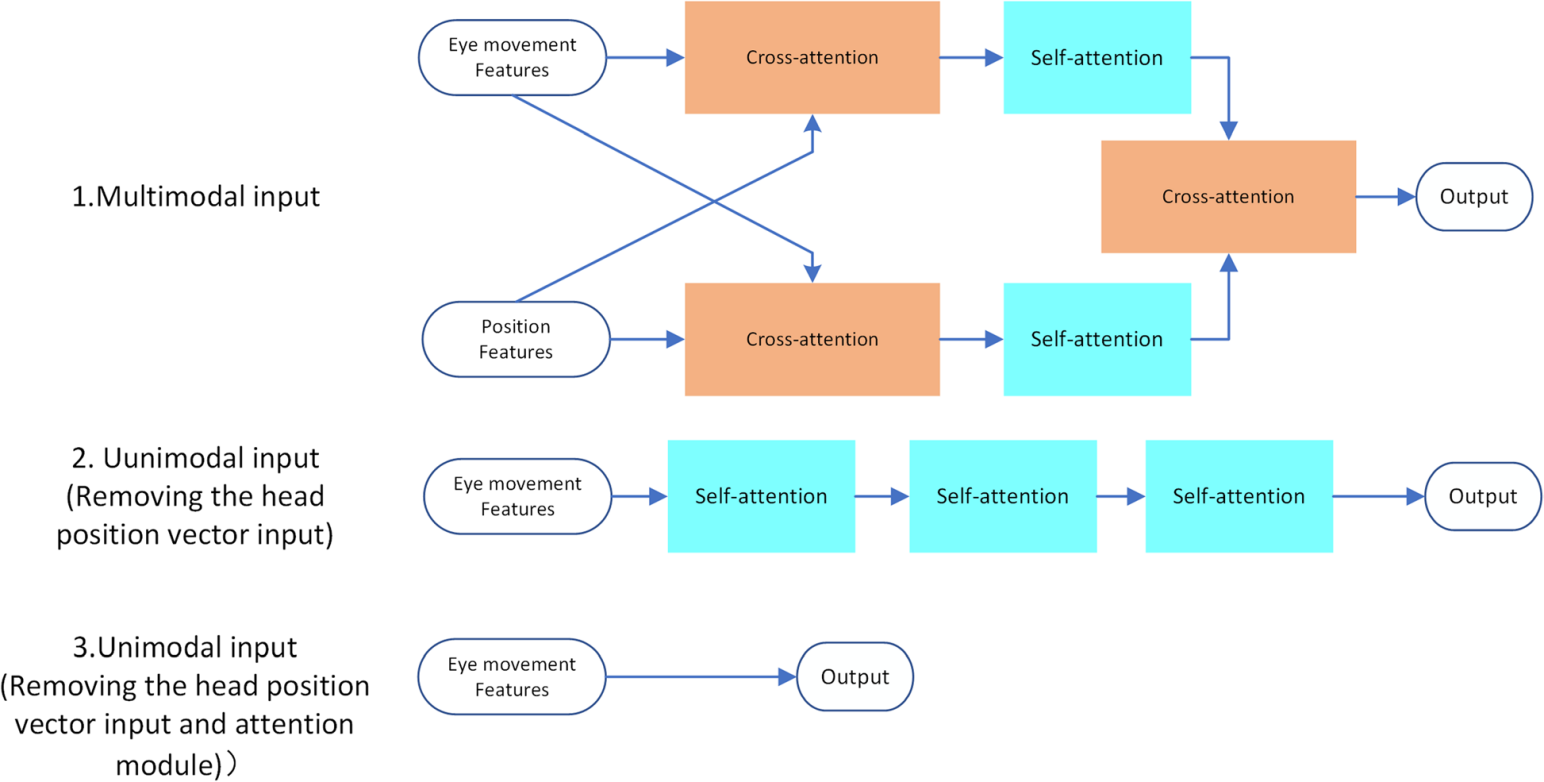
Feature fusion part of the model under different modal inputs. Under unimodal input, the attention module is retained, and the original cross-attention module becomes the self-attention module; the attention module is removed and output directly after extracting the eye-movement features

**Fig. 10:**
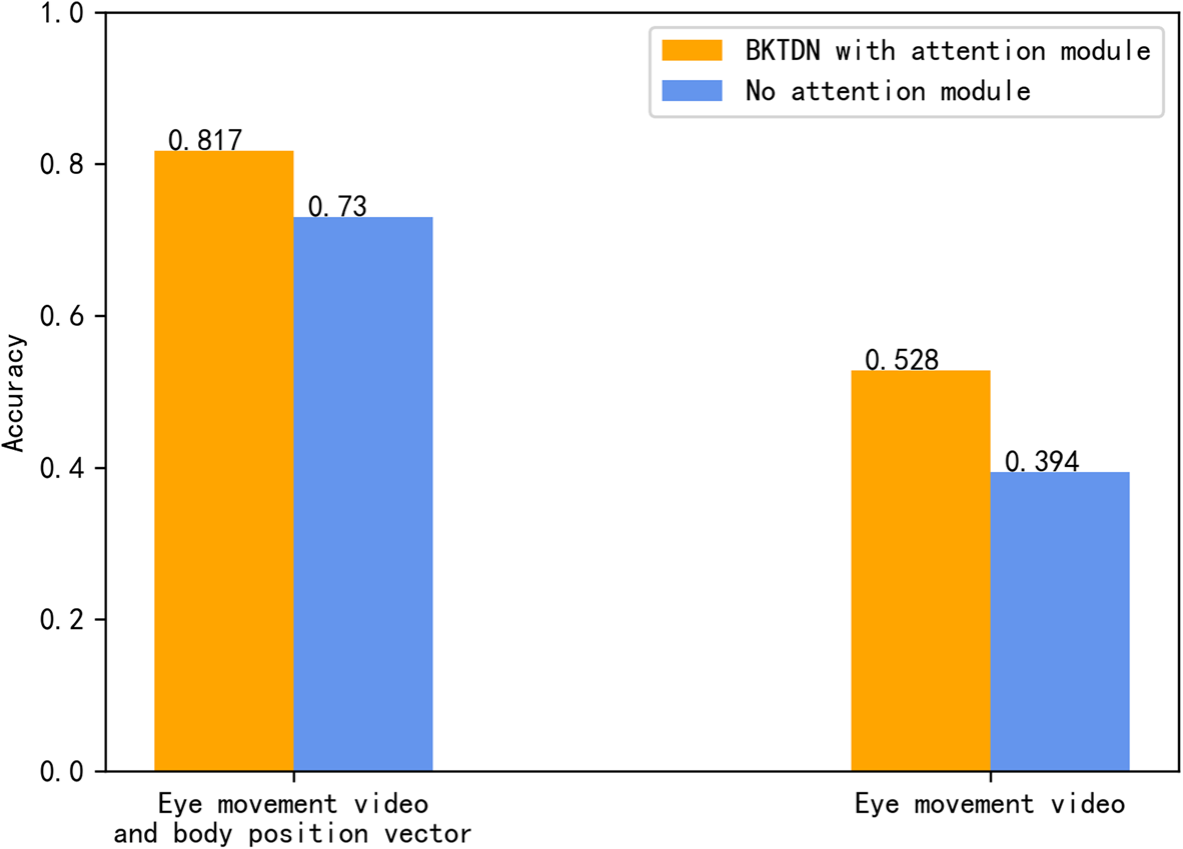
Model performance for different modal inputs

To investigate the role of position information in BPPV diagnosis, we conducted a comparison experiment between an unimodal input model and a multimodal model. As shown in Fig. 9 we tested two types of unimodal input models: (1) a model with the self-encoder removed, leaving only the attention module, and all cross-attention inputs changed to the eye-movement feature vector, which is equivalent to a self-attention mechanism; (2) a model with all parts related to the head position vector directly removed, both the self-encoder and cross-attention module were removed, and only the eye-movement feature vector was used for classification. We kept training parameters consistent for both unimodal and multimodal models and randomly initialized the modal parameters for five training sessions, taking the average of the five results on the test set for each. As shown in Fig. 10, the results indicate that the unimodal model relying solely on eye-movement video experiences a sharp decrease in accuracy from 81.7% to 52.8%. Further removing the attention module causes a further decline to 39.4%. This highlights the crucial role of head position information in BPPV diagnosis and confirms the effectiveness of our model design for processing such information. Our model incorporates a head position vector self-encoder and cross-attention module for information cross-fusion, effectively learning the spatial information within the head position vector and incorporating both the oculomotor and head position features when making a BPPV diagnosis. These findings are consistent with medical principles of diagnosis and demonstrate the importance of incorporating position information in our model.

#### Feature fusion

In the feature fusion part of the model, we made different attempts during our research: the eye movement feature vector and head position feature vector obtained from the eye movement video and head position vector in 6 different head positions were directly spliced, after which they were directly input to the classifier; trainable weights were added and the eye movement feature vector and head position feature vector were weighted and summed to obtain the synthetic feature vector, which was input to the classifier; the introduction of the cross-attention mechanism is introduced as a feature fusion module, and the eye-movement feature vector and the head position feature vector are directly used as inputs to obtain the synthetic feature vector. The final experimental results show that the model performs best with the introduction of the cross-attention mechanism.

**Fig. 11:**
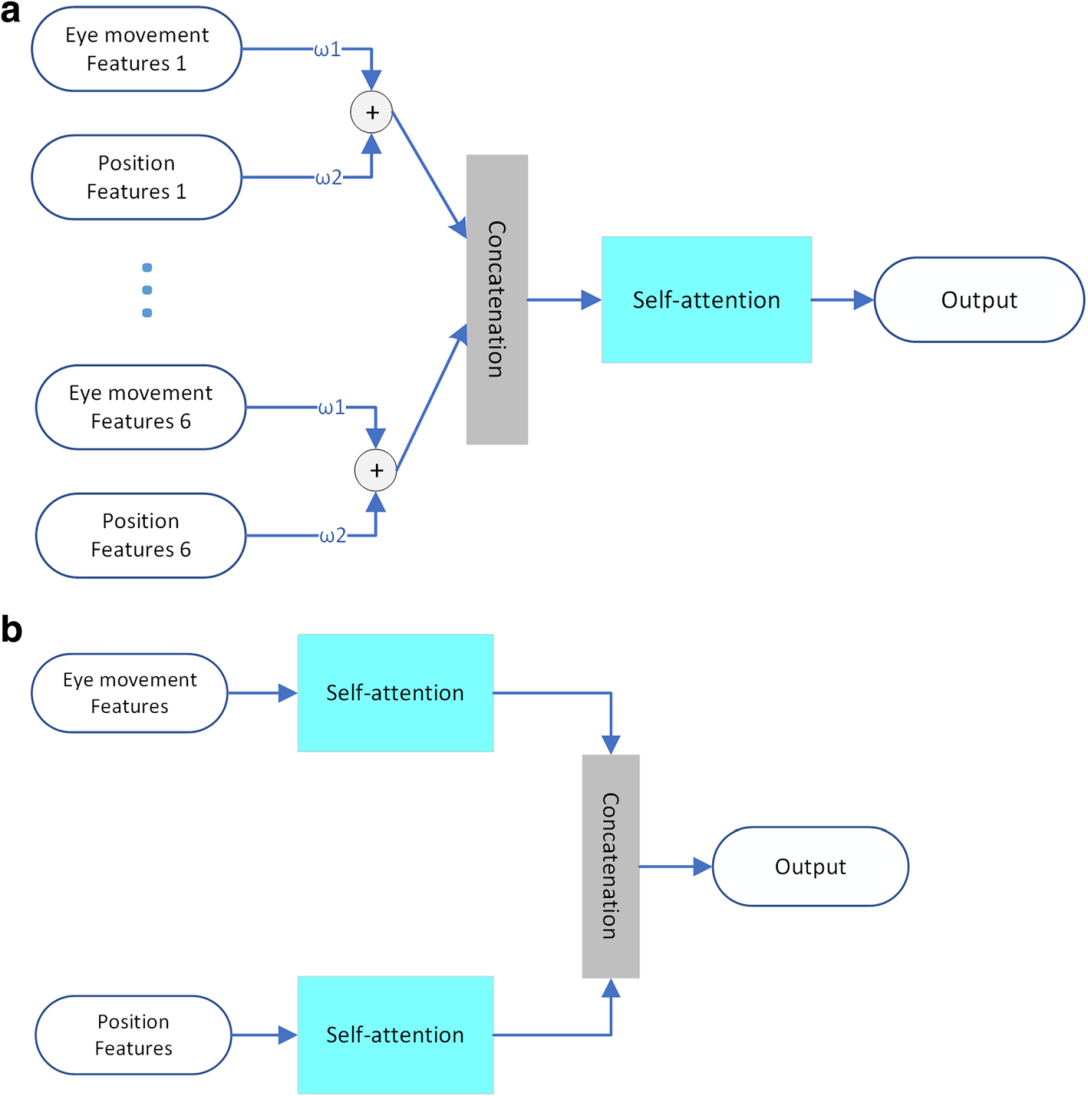
Schematic diagram of feature fusion scheme. **a**: Weighted summation + self-attention module feature fusion scheme **b**: Self-attentive feature fusion scheme

**Fig. 12:**
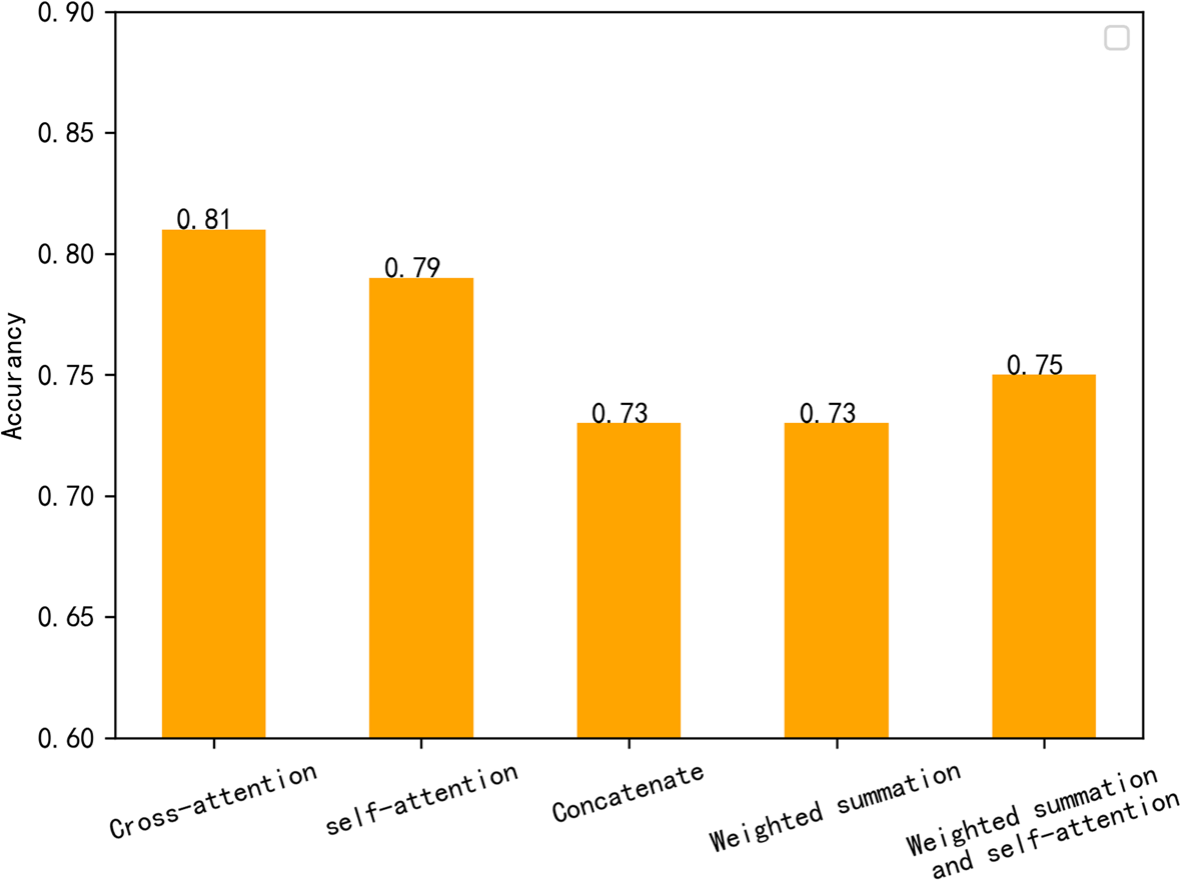
Comparison of the performance of different feature fusion schemes

To investigate the necessity of the cross-attention mechanism for model feature information fusion, we conducted a comparison experiment on the cross-attention mechanism. We first changed the cross-attention module into a self-attention module by directly removing the input of the head position feature vector of the model cross-attention module and keeping only the input of the eye movement feature vector (as shown in Fig. 11). In addition, we added the feature fusion methods taken in previous studies: direct splicing synthesis and weighted summation schemes to the comparison experiments on the cross-attention mechanism. First, the direct splicing synthesis of the two feature vectors directly removes the entire cross-attention module, and the model retains only the TDN head. TDN directly splices the eye-movement feature vector and the head position feature vector into the final FC layer. Next, we add trainable weights, the two feature vectors are weighted and summed, and the model goes through the training process to adjust the weights to obtain the synthetic feature vectors. The experimental results are shown in Fig. 12.

Comparative experimental results demonstrate that the inclusion of the cross-attention module is highly conducive to information fusion and the construction of internal associations of information. Strikingly, removing the entire cross-attention module leads to a considerable drop in the model’s performance by almost 10%, resulting in an accuracy of only 71.1% on the test set and 73.0% in the weighted summation scheme. These findings underscore the significant improvement on the information fusion ability of the model when the cross-attention module is incorporated. Interestingly, eliminating the cross-inputs of the head position feature vector and eye movement feature vector of the cross-attention model in the self-attention + splicing synthesis scheme only results in a minor 2% decrease in accuracy compared to the cross-attention scheme. However, the accuracy of the final result in the weighted summation scheme is significantly boosted by 3.9% when the self-attention mechanism is introduced. This suggests that the self-attention mechanism has a more substantial impact on the model’s ability to construct internal correlations of information and extract key information related to BPPV from the eye movement feature vector and head position feature vector.

#### Large convolution kernel

**Fig. 13:**
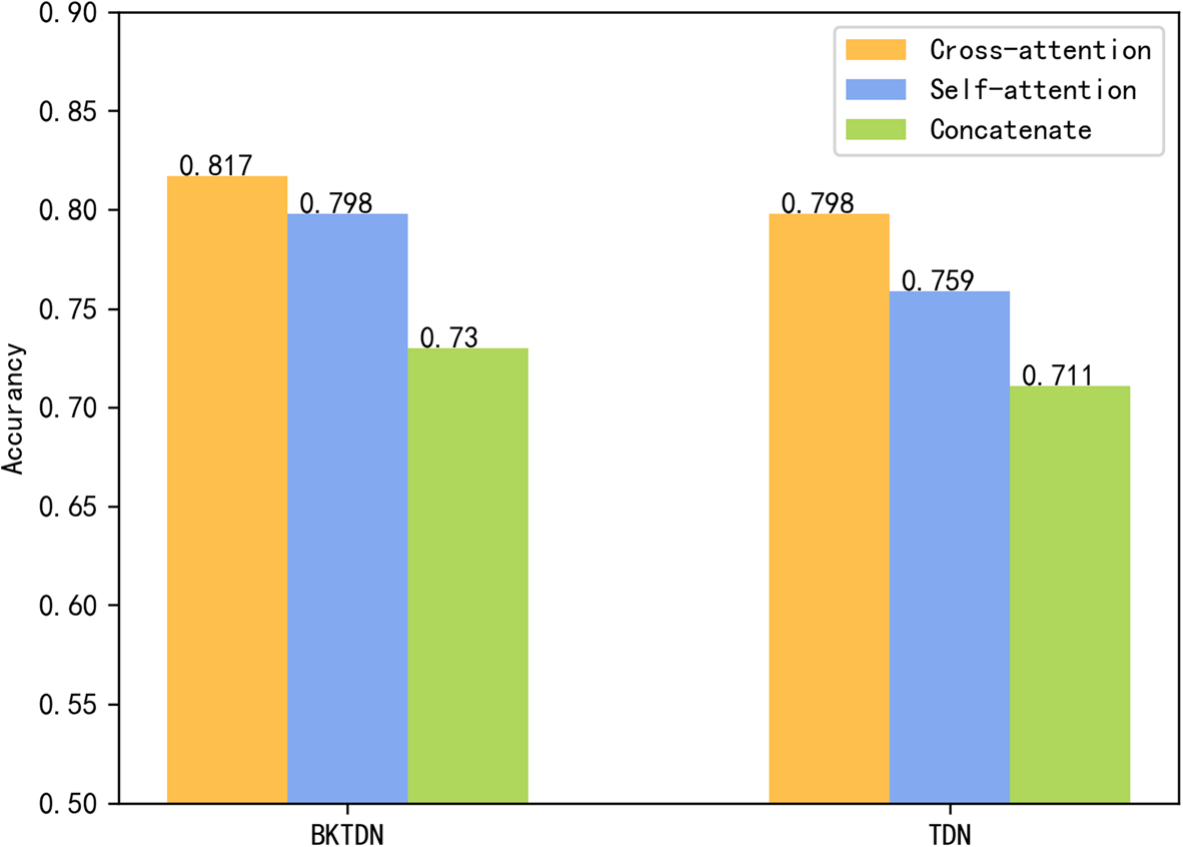
BKTDN and TDN performances comparison

In recent research, it has been demonstrated that large convolution kernels exhibit stronger performance than traditional 3 $\times$ 3 convolutions in CNNs [26, 27]. Considering the significant proportion of the eye region in the BPPV dataset’s image area, we employ a large convolution kernel to replace the original convolution kernel in the TDN structure. This is done to leverage the larger receptive field of the large convolution kernel and improve the TDN’s ability to extract features of eye movements. To verify the effectiveness of the large convolution kernel design, we conduct comparison experiments between the large convolution TDN and the original TDN with all TDN structures replaced with 3 $\times$ 3 convolution kernels. As shown in Fig. 13, the TDN structure replaced with large convolution kernels in the TDN model exhibits varying degrees of accuracy improvement in the test set, regardless of the structure. This provides evidence that our large convolution kernel design is effective in enhancing the model’s ability to extract features of eye movements.

#### Disease classification performance

In order to comprehensively validate the superior performance of the BKTDN model for BBPV diagnosis, we used classic time-series classification models, including ResNet [35], MSCNN [36], MLSTM [37], InceptionTime [38], and Rocket [39], as control groups. We trained these control networks with the same specifications as the BKTDN model. Training hyperparameters were set with 1000 training epochs, a batch size of 20, and the Adam optimizer was chosen as the loss optimizer. The learning rate followed a sine decay strategy, starting at 0.01. For each BBPV type, 80% of the data was used for training, and the remaining 20% was used for testing.

Four commonly used performance metrics for model classification were selected as quantitative standards for testing results: Accuracy, Precision, Sensitivity, and Specificity. These metrics are calculated based on the quantities of the four possible classification outcomes: 1. True Positive (TP): a positive example correctly predicted as positive; 2. True Negative (TN): a negative example correctly predicted as negative; 3. False Positive (FP): a negative example incorrectly predicted as positive; 4. False Negative (FN): a positive example incorrectly predicted as negative. From these four classification outcomes, the corresponding classification performance metrics can be calculated.

13 $$\begin{aligned} \left\{ \begin{array}{l} Accuracy=\frac{TP+TN }{TP+TN+FP+FN } \\ Precision=\frac{TP }{TP+FP} \\ Sensitivity=\frac{TP }{TP+FN} \\ Specificity=\frac{TN }{TN+FP} \\ \end{array}\right. \end{aligned}$$

**Fig. 14:**
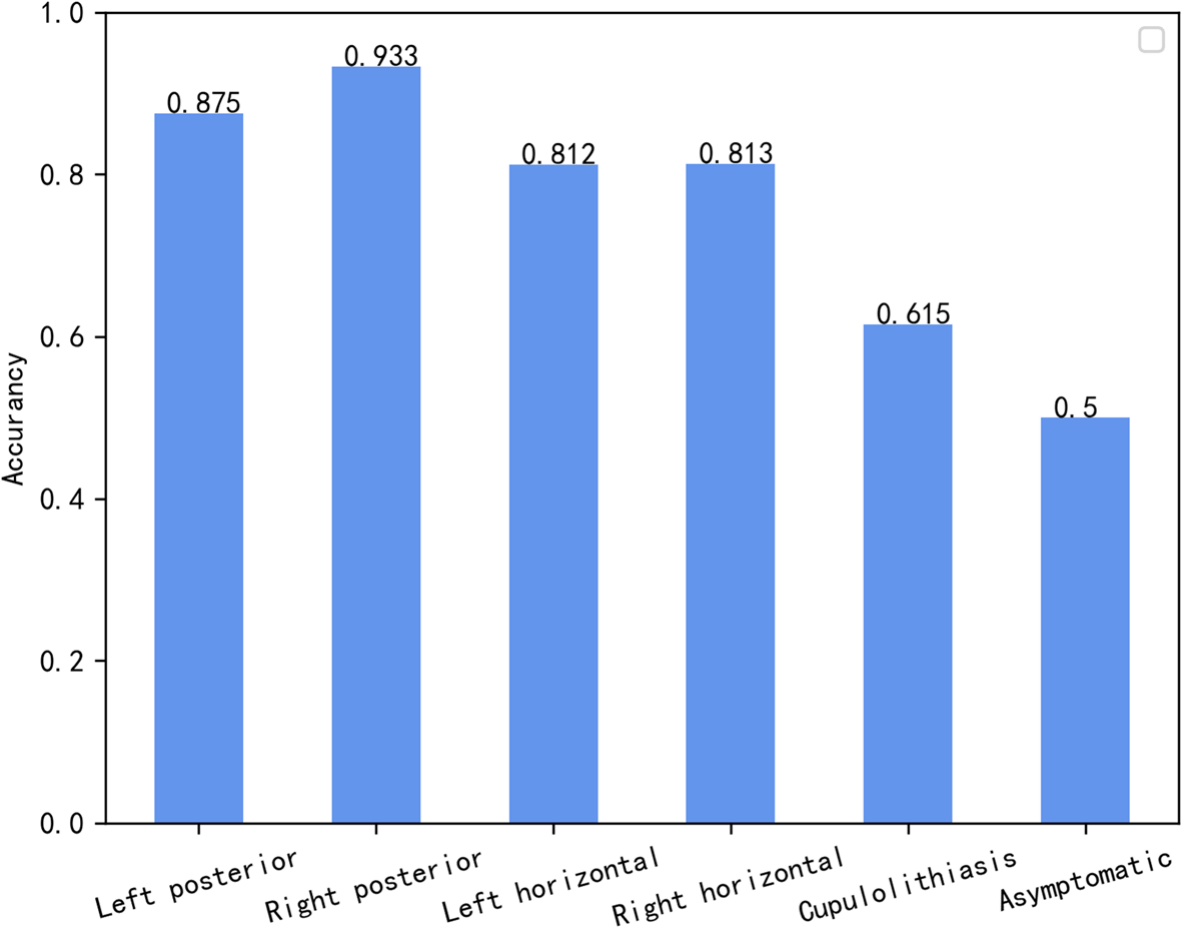
Performance of models in different types of diseases

The trained models were tested for performance on the test dataset, and the experimental results are shown in Table 3. It can be observed that BKTDN achieved the best classification results in the comparative experiments with different models. Each metric showed significant improvements compared to the control models, with Accuracy (Acc) reaching as high as 81.7%, which is 10.5% higher than the ResNet. This once again confirms that the model designed for BBPV in this paper has a strong competitive edge, even when compared to these well-established high-performance models. Additionally, in order to explore the performance of BKTDN across different BBPV disease types, We tested the diagnostic performance of the model for each type of BPPV in the dataset separately for that type of BPPV. The results are shown in Fig. 14. After comparing the test results, we found that the accuracy of the test results was higher in the posterior and horizontal semicircular canal BPPV, but lower for the cupulolithiasis type of BPPV and the healed or asymptomatic type.

**Table 3:** Performance comparison between classical models in the BBPV dataset

Models	Accuracy (%)	Precision (%)	Sensitivity (%)	Specificity (%)
ResNet	71.2	73.9	90.6	94.0
MSCNN	75.0	77.8	91.9	94.9
MLSTM	72.1	74.4	90.9	94.2
Inceptiontime	74.0	75.8	91.7	94.9
Rocket	75.0	76.7	92.0	95.0
**BKTDN**	**81.7**	**82.1**	**94.1**	**96.5**

## Discussion

We have verified the indispensability of head position information in the diagnostic model of BPPV, as well as the efficacy of utilizing a large convolution kernel to improve model performance. Moreover, our experimental results have demonstrated the superiority of cross-attention module as a feature fusion scheme in the diagnostic model. However, despite our efforts, the classification accuracy of individual BPPV types fell below the desired threshold during testing, thus compromising the overall model performance on the given dataset. Through previous observation of the physician’s diagnostic process in the field and communication with the physician to understand, we believe the possible reasons are

1. The cupulolithiasis type of BPPV usually has a longer period of vertigo or nystagmus during the diagnosis process, which is not triggered by nystagmus in some positions, and during the diagnosis process, the physician will repeatedly ask the patient whether he or she has symptoms of vertigo, but the process of asking about vertigo is not recorded, but vertigo is also an important basis for the diagnosis of this type of condition. We collected the data set at a later stage due to the lack of vertigo records, so we only used the eye movement video and head position information as the classification basis of the model during the model design. And our understanding of cases of BPPV with cupulolithiasis is limited. We have not yet discussed issues related to the affected canals with medical professionals. Exploring this particular category of cases will be a focus of our future research efforts, and it may necessitate the development of a dedicated classification scheme.
2. At the same time, for healed patients, doctors will also perform the steps of therapeutic reset during the diagnosis process, and during the reset process, patients may also have some symptoms of nystagmus, which will be recorded in the video, but when to start the process of therapeutic reset, these are also not recorded in our later collection of videos.
3. For patients such as cupulolithiasis, or patients cured in the course of treatment, the doctor’s observation in some positions is longer than 48s, but in order to ensure the input shape is consistent and to prevent the data volume of BPPV’s eye movement video dataset from being too large, we only intercept the eye movement video in each position for a maximum length of 48s during the preliminary processing, resulting in some important information after 48s not being intercepted This resulted in some important information after 48s not being captured.

Therefore, the model showed less precision and less accuracy for these symptoms with certain missing information such as cupulolithiasis BPPV. The model performs better for symptoms such as posterior semicircular canal where nystagmus symptoms are more transient and obvious.

Based on the aforementioned limitations and challenges, there are several avenues for future research that we plan to pursue. Firstly, to improve the accuracy of the diagnostic model for BPPV, we will explore novel data collection techniques that can document important diagnostic information such as vertigo symptoms and the timing of therapeutic reset. Secondly, we will investigate the use of advanced machine learning techniques to better capture the temporal dynamics of BPPV symptoms over extended periods of time, thereby enabling more accurate classification of individual types of BPPV. Thirdly, we will examine the feasibility of using a combination of different types of data, such as eye movement video, head position information, and audio recordings of physician-patient interactions, to provide a more comprehensive basis for the diagnosis of BPPV. Furthermore, based on the advice of specialized medical professionals, we have learned that, in order to investigate the affected ocular pathways, examining the rotational movement of the eyeballs is more critical than assessing their linear motion. The rotational movement of the eyeball, which constitutes a 3D spherical motion, involves considerations related to video clarity, experimental equipment functionality, and extraction algorithms. This will be one of the directions we aim to explore in our future research endeavors.

Overall, we believe that these efforts will enhance our understanding of BPPV and improve the accuracy and effectiveness of diagnostic models, ultimately leading to better patient outcomes and improved quality of care.

## Conclusion

In this study, we propose a novel multimodal diagnostic model for BPPV, which combines head position and eye movement video data to improve the accuracy and effectiveness of diagnosis. Our model is based on a deep learning architecture that leverages both head position vectors and eye movement videos as inputs. To optimize the model’s performance, we have made several enhancements to our video understanding model, TDN, including the introduction of a large convolutional kernel to extract relevant information from eye-movement videos. Additionally, we have developed a head position vector self-encoder and pre-training method to extract spatial features from head position vectors and transform them into feature vectors containing spatial information. Finally, we have introduced a cross-attention mechanism to perform effective feature fusion of the output feature vectors from different modalities. Our experimental results demonstrate that the proposed multimodal model performs best in diagnosing various types of BPPV compared to other classical time-series classification models. However, due to limitations in the available dataset, certain critical diagnostic information was not captured, leading to suboptimal accuracy in some BPPV types. And our research on cupulolithiasis-type BPPV is not sufficiently in-depth. In the experiments, we did not specify which semicircular canal was affected (horizontal canal, posterior canal, or anterior canal) and did not mention the affected side. Addressing these challenges will be a key focus of our future research efforts.

## Data Availability

The datasets used in this study belong to the Second Affiliated Hospital of the Army Medical University,the authors do not have the authority to disclose these data,but they are available from the corresponding author with content of the ethics committee.
